# Transovarial Transmission of a Plant Virus Is Mediated by Vitellogenin of Its Insect Vector

**DOI:** 10.1371/journal.ppat.1003949

**Published:** 2014-03-06

**Authors:** Yan Huo, Wenwen Liu, Fujie Zhang, Xiaoying Chen, Li Li, Qifei Liu, Yijun Zhou, Taiyun Wei, Rongxiang Fang, Xifeng Wang

**Affiliations:** 1 State Key Laboratory of Plant Genomics, Institute of Microbiology, Chinese Academy of Sciences, Beijing, China; 2 State Key Laboratory for Biology of Plant Diseases and Insect Pests, Institute of Plant Protection, Chinese Academy of Agricultural Sciences, Beijing, China; 3 Fujian Province Key Laboratory of Plant Virology, Institute of Plant Virology, Fujian Agriculture and Forestry University, Fuzhou, Fujian, China; 4 National Plant Gene Research Center, Beijing, China; 5 Graduate School of the Chinese Academy of Sciences, Beijing, China; 6 Jiangsu Academy of Agricultural Sciences, Nanjing, China; University of Kentucky, United States of America

## Abstract

Most plant viruses are transmitted by hemipteroid insects. Some viruses can be transmitted from female parent to offspring usually through eggs, but the mechanism of this transovarial transmission remains unclear. *Rice stripe virus* (RSV), a *Tenuivirus*, transmitted mainly by the small brown planthopper (*Laodelphax striatellus*), is also spread to the offspring through the eggs. Here, we used the RSV–planthopper system as a model to investigate the mechanism of transovarial transmission and demonstrated the central role of vitellogenin (Vg) of *L. striatellus* in the process of virus transmission into the eggs. Our data showed Vg can bind to pc3 *in vivo* and *in vitro* and colocalize in the germarium. RSV filamentous ribonucleoprotein particles (RNPs) only accumulated in the terminal filaments and pedicel areas prior to Vg expression and was not present in the germarium until Vg was expressed, where RSV RNPs and Vg had colocalized. Observations by immunoelectron microscopy (IEM) also indicated that these two proteins colocalized in nurse cells. Knockdown of Vg expression due to RNA interference resulted in inhibition of the invasion of ovarioles by RSV. Together, the data obtained indicated that RSV RNPs may enter the nurse cell of the germarium via endocytosis through binding with Vg. Finally, the virus enters the oocytes through nutritive cords, using the same route as for Vg transport. Our results show that the Vg of *L. striatellus* played a critical role in transovarial transmission of RSV and shows how viruses can use existing transovarial transportation systems in insect vectors for their own purposes.

## Author Summary

Numerous parasites including viruses, bacteria, and microsporidia can be maternally transmitted, with the parasite passing from mother to offspring, usually through eggs. However, the process of the parasites spreading into eggs from primarily infected tissues and the factors that mediate this process in live hosts or vectors are unknown due to the lack of useful tools. Here, we used several techniques to investigate the molecular mechanisms of transovarial transmission of *Rice stripe virus* (RSV), a plant virus belonging to the genus *Tenuivirus*, by its insect vector (*Laodelphax striatellus*). We found that the nucleocapsid protein of RSV bound to insect's vitellogenin (Vg) *in vitro* and *in vivo*. We also found that RSV invaded the egg tubes of the ovariole until Vg is highly expressed, then colocalized with Vg in the germarium. When Vg expression was knocked down due to RNA interference, the invasion of ovarioles by RSV decreased largely. Our study provides new insights into the transovarial transmission of an important viral pathogen that uses existing transovarial transportation systems in insect vectors to invade eggs.

## Introduction

Many viruses, especially plant viruses, are transmitted by insects. Viruses can be transmitted horizontally from individual to individual and vertically from parents to offspring [1]. More than 200 plant viruses are transmitted by specific arthropod vectors; some are retained for a few hours or days after acquisition and others for up to the life of the insect, i.e., in a persistent circulative or persistent-propagative manner [2]. Plant viruses such as tospoviruses, rhabdoviruses, marafiviruses, tenuiviruses, and reoviruses are transmitted by their respective insect vectors in a persistent-propagative manner [1]. The persistent-propagative viruses can replicate in different organs of insect vectors, and some of these viruses can be transmitted vertically from female parent to offspring in a transovarial manner. The transmission process is a critical step for every virus because it controls dispersal in space and time, thus directly influencing virus ecology and disease epidemiology.

During the general transmission process of persistent-propagative viruses, virions first accumulate in the midgut lumen of the insect and then pass through the midgut wall to disperse into the hemolymph or nervous tissues, then into the salivary gland from where the viruses can be introduced back into the host plant during insect feeding [2]–[5]. Furthermore, some viruses, including plant rhabdoviruses, tenuiviruses, and plant reoviruses, can spread into the ovary from where the virions can be transmitted to the offspring for many generations [6]–[8]. For example, *Rice stripe virus* (RSV), a *Tenuivirus*, can be transmitted transovarially to the progeny of planthopper vectors for 40 generations. *Rice dwarf virus* (RDV), a *Phytoreovirus*, has been demonstrated to be transmitted transovarially in *Nephotettix cincticeps* and maintained through generations of progeny for 6 years [9].

In addition to viruses, other microbes including bacteria, microsporidia and fungi can be maternally transmitted by arthropods [10]. These microbes have evolved many strategies to spread through host populations. Maternal transmission of endoparasitic microbes in an insect host is possibly related to vitellogenin (Vg), a female-specific protein synthesized mainly by the fat body and secreted into hemolymph, from where it is absorbed by the growing oocytes via receptor-mediated endocytosis [11], [12]. Transovarially transmitted yeast-like symbionts (YLSs) in brown planthoppers are wrapped in Vg outside the ovary [13]. Moreover, the ZAM virus in *Drosophila melanogaster* and the *Babesia* parasite in *Haemaphysaalis longicornis* are dependent on the Vg receptor when transmitted into the oocyte [14]–[16]. However, the mechanism by which plant viruses spread into the insect ovary has rarely been reported, and vector proteins that are involved in overcoming the barriers to transovarial transmission of viruses within their insect vectors have not been directly characterized or even precisely located. This question is of major importance because identification of putative components could lead to new strategies to combat vertical viral spread.

**Figure 1:**
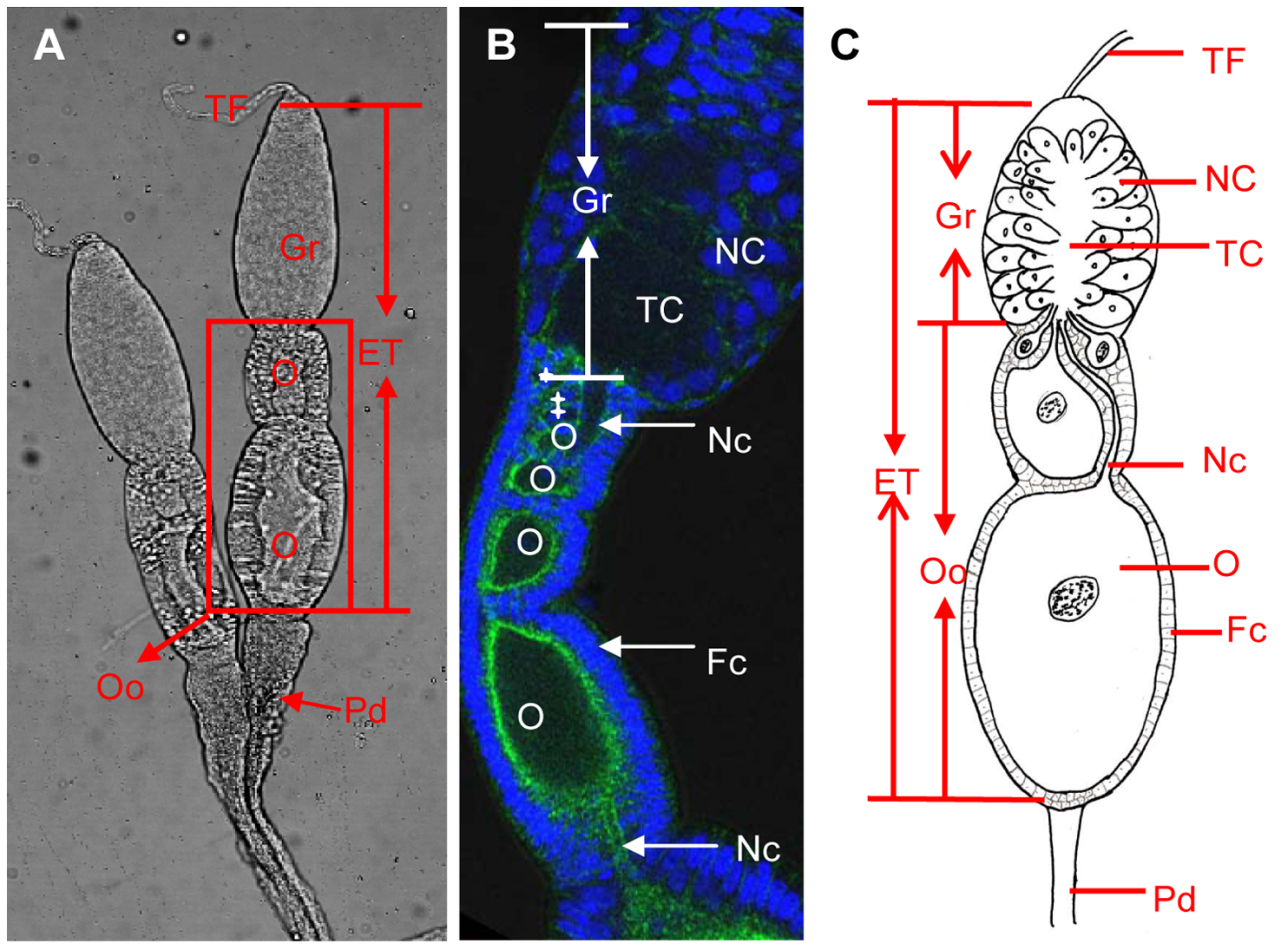
Ovary structure of *Laodelphax striatellus*. a: The ovariole of *L. striatellus* is composed of a terminal filament on the tip, the egg tube in the middle, and a pedicel at the bottom. The germarium is at the tip of the egg tube; ooecia, just below the germarium, are composed of oocytes and follicular cells. b: Ovarioles at the previtellogenesis stage were stained with DAPI for DNA (blue) and FITC-phalloidin for actin (green). The cell-free trophic core and several oocytes (white stars) are located in the middle and the base of the germarium, respectively. c: Schematic drawing of typical telotrophic meroistic ovariole. Nurse cells in the germarium radially arranged around the trophic core in the middle. The nutritive cord connects the trophic core and oocytes. ET: egg tube, Fc: follicular cell, Gr: germarium, NC: nurse cell, Nc: nutritive cord, O: oocyte, Pd: pedicel, TF: terminal filament, TC: trophic core.

Here, we used the RSV–planthopper system as a model to investigate the mechanism of transovarial transmission of a plant virus in an insect vector. RSV is transmitted by the *L. striatellus* in a persistent-propagative manner and has caused serious yield losses in rice production in East Asia [17]. *Laodelphax striatellus* is an important agricultural pest, not only infesting cereal plants, but also as a vector of several viruses. It transmits RSV and *Rice black-streaked dwarf virus* in a persistent-propagative manner, but only RSV is transmitted in a transovarial manner [18]. RSV can effectively spread into the ovarioles of *L. striatellus*. Insect ovarioles, a functional unit of the ovary, are divided into three types according to the mode of nutritional intake of the oocyte, i.e., panoistic ovarioles, polytrophic meroistic ovarioles, and telotrophic meroistic ovarioles [19]. The ovarioles of *L. striatellus* are the telotrophic meroistic type, which consists of a polarized tube with a germarium, comprising a cluster of nurse cells, at its anterior end [17] (Figure 1a). All nurse cells are radially arranged around and connected to the central trophic core; oocytes are arranged on the base of the germarium previtellogenesis; and nutrients are transported from the nurse cells to the developing oocytes through nutritive cords that connect nurse cells and oocytes [20]–[23] (Figure 1b, c).

RSV has filamentous ribonucleoprotein particles (RNPs), which contain four single-stranded RNAs, and the major nucleocapsid protein (pc3) encoded by the ORF at the 5′ half of the viral complementary RNA3 [7]. Thus, pc3 is considered the key viral component for specifically interacting with the vector components and may play an important role in RSV transovarial transmission. Here, we used various technologies, yeast two-hybrid system, chemiluminescent coimmunoprecipitation, glutathione-*S* transferase pull-down assay, immunofluorescence laser scanning confocal microscopy (iCLSM), immunoelectron microscopy and RNA interference experiments to identify the essential role of Vg in the transovarial transmission of RSV. Our results provided evidence that the interaction of RSV pc3 and Vg of *L. striatellus* might be a critical step in the transovarial transmission of RSV.

## Results

### Isolation and characterization of the *L. striatellus* proteins that interact with RSV pc3

RSV pc3 was used as bait to screen the *L. striatellus* cDNA library by a yeast two-hybrid system, and a 375-bp DNA fragment encoding the open reading frame of Vg was isolated. Cotransformation assays confirmed that RSV pc3 interacts with Vg protein fragments in yeast (Figure S1a, b). In three independent chemiluminescent coimmunoprecipitation assays, we then confirmed the *in vitro* interaction between pc3 and Vg (fragment obtained from yeast two-hybrid screens) by coexpression in HEK 293FT cells (Figure S1c, d).

Based on previously published sequences of *Vg* mRNA fragments of *L. striatellus* [24], we used PCR, 5′ RACE and 3′ RACE methods to amplify and clone the full-length *Vg* cDNAs. Two full lengths of *Vg* cDNA were obtained with 6415 and 6265 bp (GenBank accessions KC469580 and KC469581, respectively). The 6415-bp cDNA has a 6135-bp open reading frame (ORF) coding for a deduced 2045-amino-acid (aa) protein; the 6265-bp cDNA revealed a 6174-bp ORF coding for a deduced 2058-aa protein. The identity between the deduced amino acid sequences of these two proteins is 98.5%, and both proteins contained Vg-specific domains: the vitellogenin N (VitN) domain, an unknown function domain (DUF), and a von Willebrand domain (vWD) [25] (Figure S2).

**Figure 2:**
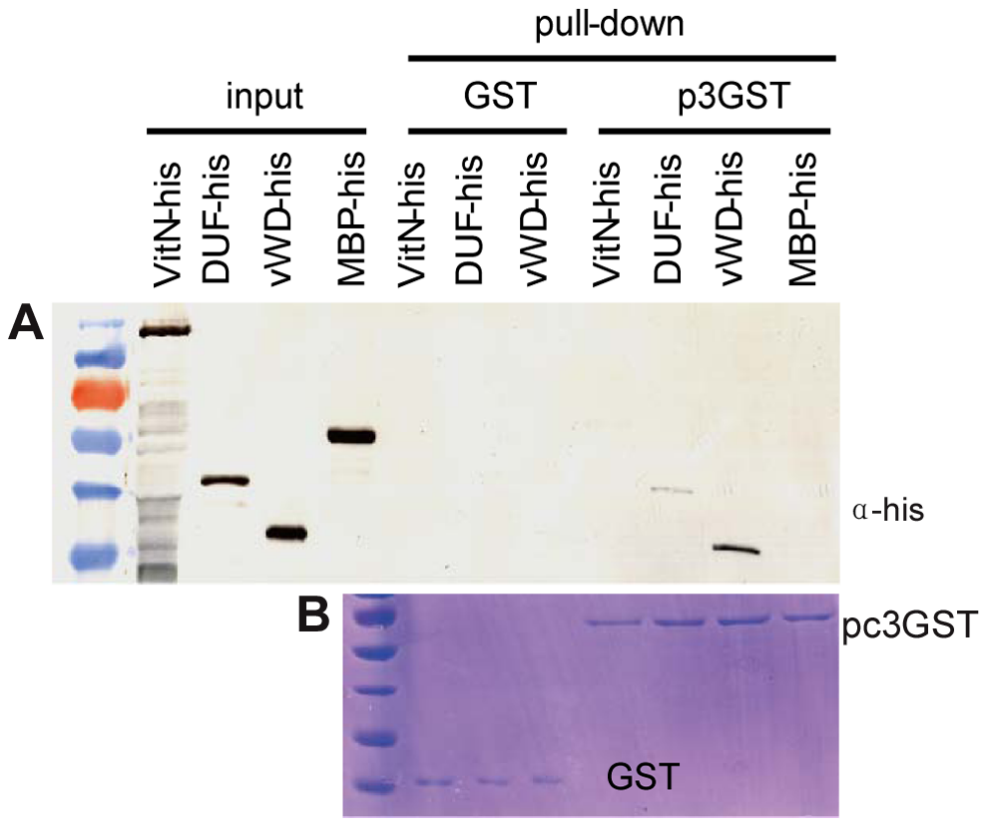
Analysis of interaction between RSV pc3 and different vitellogenin fragments *in vitr*. a: The GST pull-down assay was used to detect any interaction between RSV pc3 and Vg. RSV pc3 was fused with GST to act as a bait protein with a single GST as a control. None of the prey proteins (VitN, DUF or vWD domains) interacted with GST. VitN had no binding ability, DUF had a weak interaction, but vWD strongly interacted with pc3-GST. b: Pull-down samples were stained with Coomassie brilliant blue to ensure the equal sample loading.

To pinpoint which of these Vg domains function in binding with RSV pc3, three protein fragments, VitN, DUF and vWD of Vg (GenBank accession KC469580), were tested *in vitro* for any interaction with RSV pc3 using a pull-down assay. The results showed that the vWD and DUF domains bound to GST-fused pc3, but none bound to GST (the negative bait control). The fragment containing the vWD domain interacted more strongly with RSV pc3 than did DUF (Figure 2).

### Vg is correlated with RSV entry and spread in the insect ovariole

**Figure 3:**
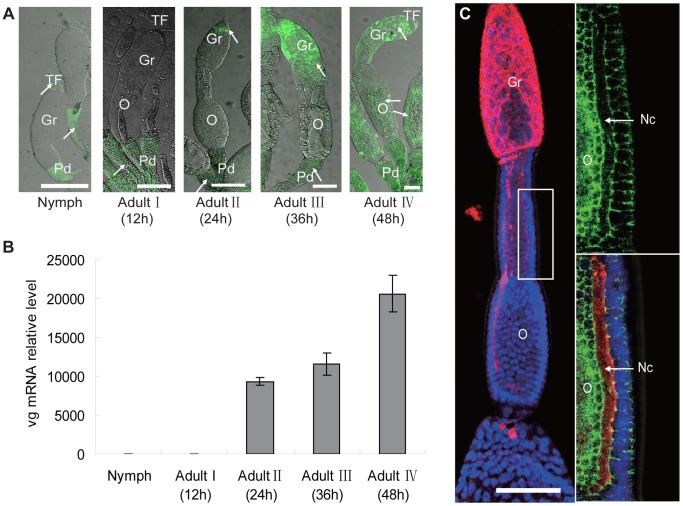
Localization of RSV in ovaries of *Laodelphax striatellus* at different stages of ovarian development. a: RSV in *L. striatellus*. In nymph and adult stage I, RSV was found only in the terminal filament and pedicel. In adult stage II, RSV invaded the tip of the gemarium; in adult stages III–IV, RSV was present throughout the germarium and in portions of the oocyte (anti-RSV monoclonal antibody is conjugated directly to Alexa Fluor 488, green;). Arrow: RSV antigens. Bar = 100 µm b: Vg was expressed at a background level in the nymph and Adult I (12 h postemergence) stages. Expression sharply increased in the Adult II stage (24 h postemergence) and continuously increased in Adult III (36 h postemergence) and IV (48 h postemergence) stages. c: RSV RNPs in the nutritive cord, which connects the germarium and oocytes. Ovarioles at Adult IV stage were treated with rhodamine-conjugated anti-RSV antibody (red) and stained with DAPI for DNA (blue), FITC- phalloidin for actin (green); bar = 100 µm. Gr: germarium, Nc: nutritive cord, O: oocyte, Pd: pedicel, TF: terminal filament.

In an examination of the distribution of RSV RNPs in the insect ovariole using monoclonal antibodies and iCLSM, we did not observe any RSV RNPs in the ovarioles of nymphs or adults at the previtellogenic stage (Adult I) when Vg was not expressed (Figure 3a, b). RSV RNPs were first detected in the ovariole germarium of adults at the early stage of vitellogenesis (Adult II) when Vg began to be expressed, then observed not only in the germarium but also in the ooecium of adults at the vitellogenic stage (Adult III and IV), when high quantities of Vg were expressed (Figure 3a, b). After RSV RNPs have successfully invaded the germarium, they would move to the nutritive cords at Adult IV stage, which connect the germarium to oocytes (Figure 3c). We also noted that RSV invaded the ovariole from its anterior side rather than the pedicel side, suggesting that a barrier between the pedicel and ooecium prevents RSV entry directly from the pedicel into the ooecium (Figure S3a).

### RSV RNPs colocalized with Vg in the germarium

**Figure 4:**
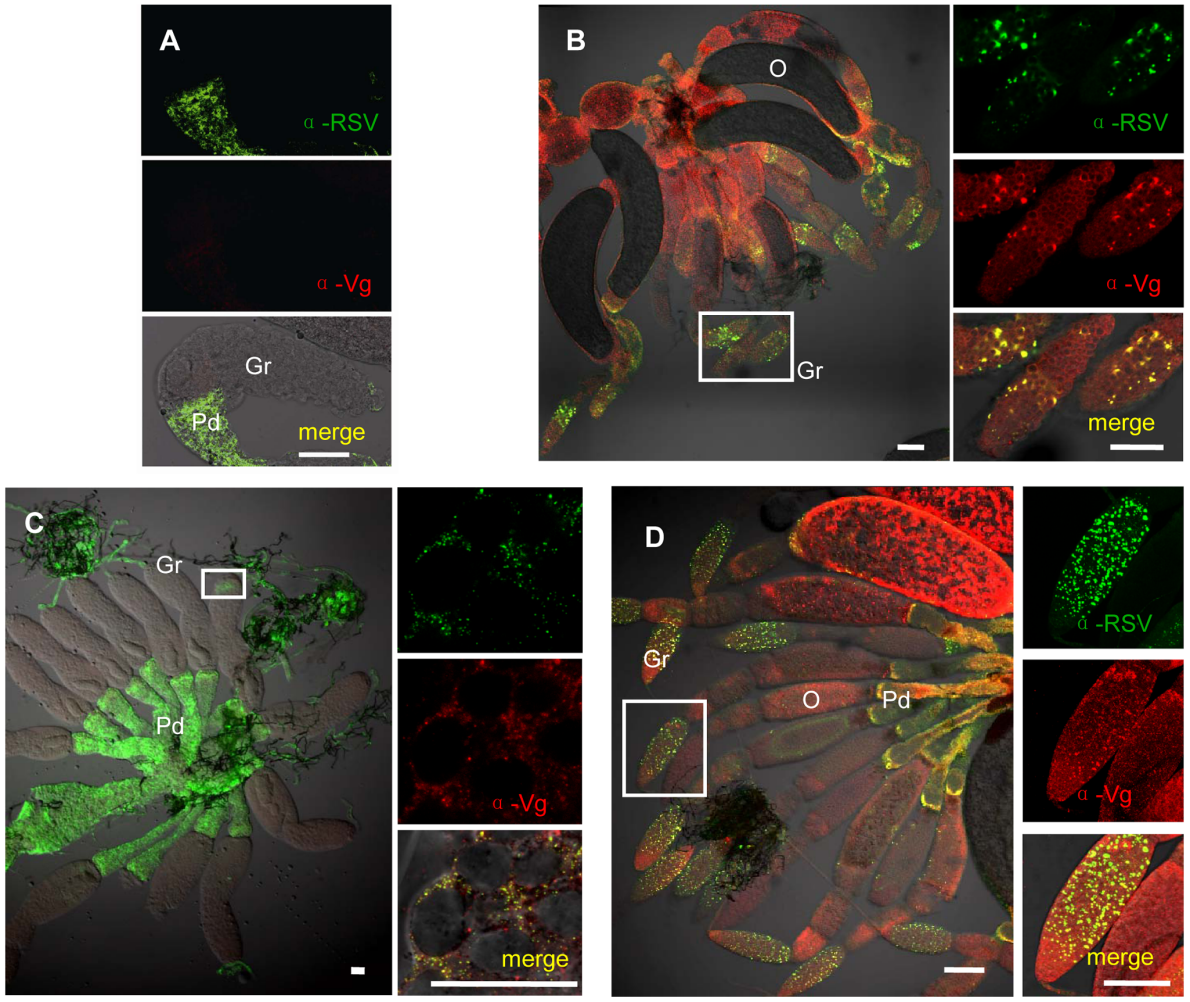
Colocalization of RSV and vitellogenin in *Laodelphax striatellus* ovaries. a: Neither RSV nor Vg antigens were detected in the germarium at previtellogenic stage. b: RSV and Vg antigen colocalization spots at vitellogenic stage (Adult III). c: RSV and Vg arenaceous colocalization spots in germ cells at early stage of vitellogenesis. d: RSV and Vg antigens colocalization spots at later vitellogenic stage (Adult IV). Anti-RSV and anti-Vg monoclonal antibodies were conjugated to Alexa Fluor 488 (green) and Alexa Fluor 594 (red) separately. A, C, D: bar = 50 µm, B: bar = 30 µm. TF: terminal filament, Gr: germarium, O: oocyte, Pd: pedicel.

**Figure 5:**
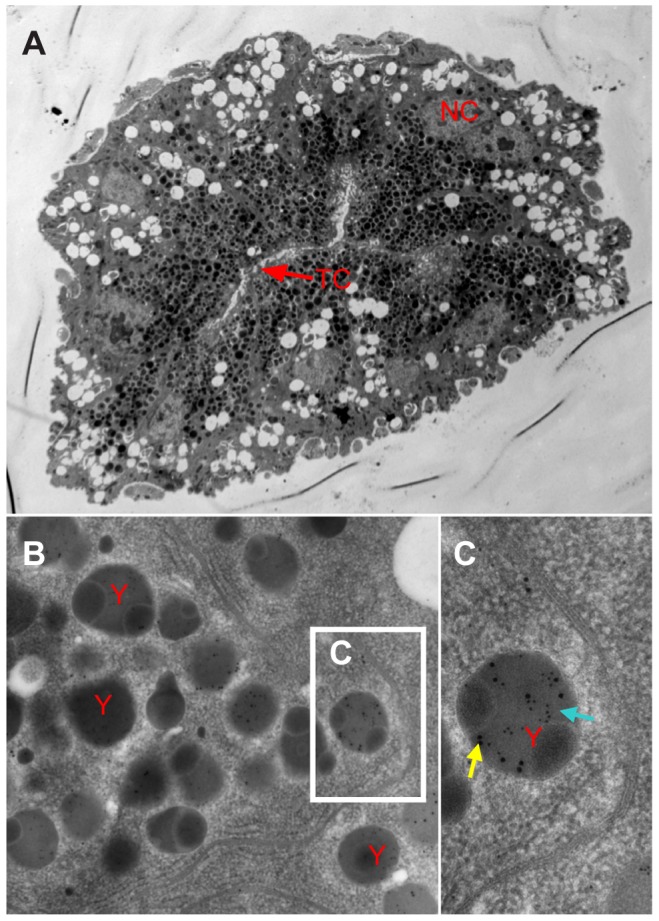
Immunoelectron micrographs showing distribution of RSV and vitellogenin in the germarium. a: Nurse cells radially arranged around the trophic core; Vg assembled in the highly electron-dense yolk granules in the nurse cells on the TC side. b: Both RSV and Vg antigens are found in the yolk granules. c: RSV (blue arrow) and Vg (yellow arrow) assembled in yolk granules. TC: trophic core; NC: nurse cells; y: yolk granule; blue arrow: 10-nm gold-conjugated goat-anti-mouse IgG against RSV used to detect virus; yellow arrow: 15-nm gold-conjugated goat-anti- rabbit IgG against Vg. A: bar = 10 µm, B: bar = 500 nm, C: bar = 100 nm.

To determine the functional role for Vg in the transovarial transmission of RSV by *L. striatellus*, we tested for colocalization of Vg and RSV RNPs again using iCLSM. Neither RSV RNP antigens nor Vg antigens were observed in the germarium ovarioles of nymph and insects at previtellogenic stage (Figures 3a and 4a). RSV antigens were only observed in the terminal filaments and pedicels of the ovarioles, as previously detected. At the early stage of vitellogenesis, RSV RNPs and Vg colocalized with each other, forming some small colocalization spots in the germarium of the ovariole (Figure 4b). With time, more colocalization spots of RSV RNPs and Vg gradually accumulated and enlarged (Figure 4c) and were finally distributed throughout the germarium of insects at vitellogenic stage (Figure 4d). However, these two proteins did not colocalize in the ooecium (Figure S3b). The location of RSV RNPs and Vg in the germarium was also detected by immunoelectron microscopy (Figure 5). In transections of the germarium of the ovariole at the vitellogenic stage, many yolk globules were observed in the nurse cells, radially arranged around a cell-free trophic core (Figure 5a). RSV RNPs and Vg accumulated in the yolk globules, with a few scattered in the cytoplasm (Figure 5b, c).

### Reducing Vg expression by RNA interference affected the invasion of germarium by RSV

**Figure 6:**
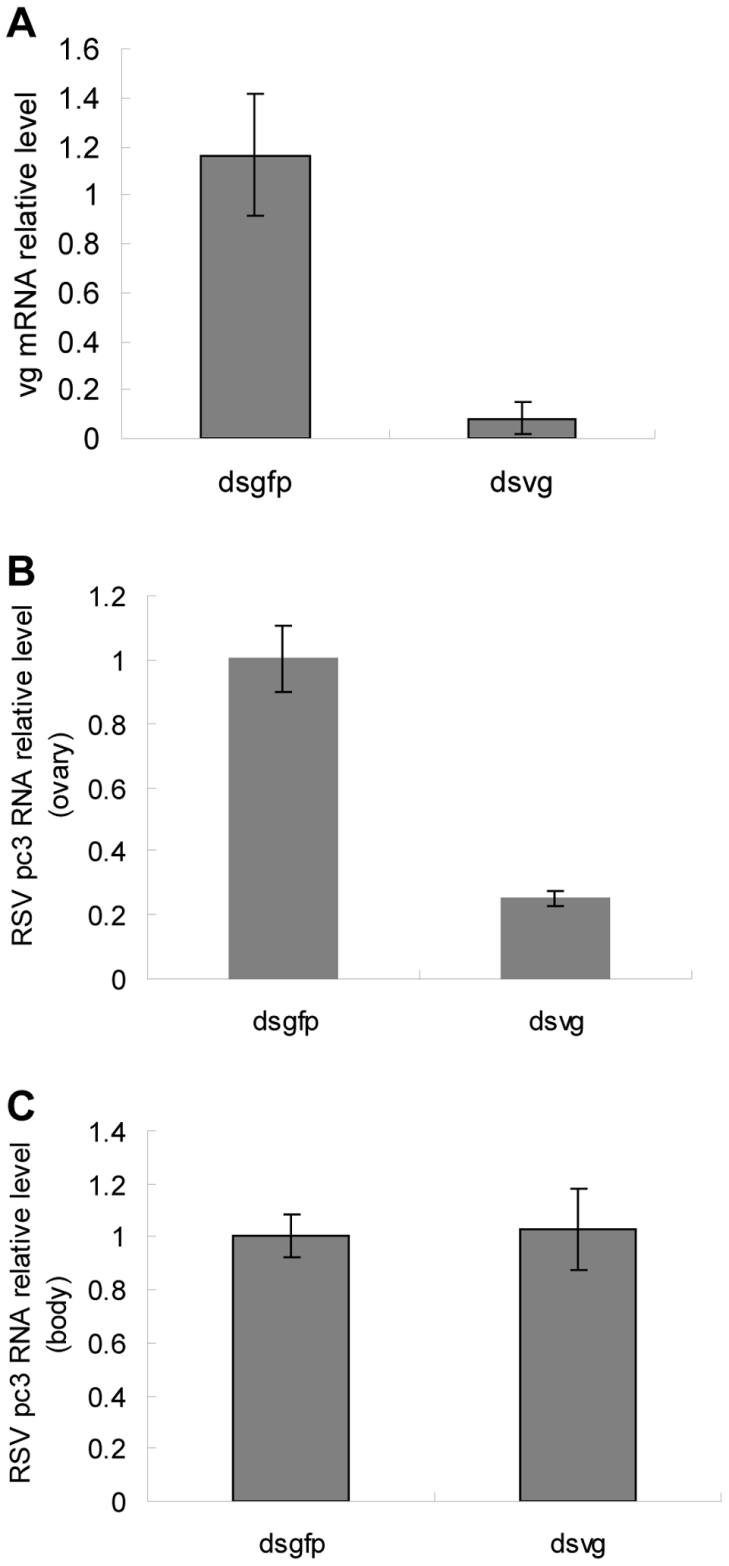
Quantitative assay of RSV in dsRNA-injected ovarioles. RSV-infected nymphs were injected with *Vg* dsRNA, with *gfp* dsRNA as a control. a: After dsRNA injection, insects that had emerged for 48 hours were selected to detect Vg expression. b: The quantity of RSV *pc3* RNA had decreased significantly by 79% in the ovarioles of viruliferous 48 h adult insects. c: RSV level in the other parts of the insect body did not decrease after injections with different dsRNAs.

Our results implied that Vg might play a role in RSV invasion of the germarium. To further test this hypothesis, we knocked down the expression of Vg using RNA interference by injecting dsRNA for *Vg* or *gfp* (dsvg or dsgfp) into the body of *L. striatellus*. Compared with those injected with dsgfp-injected insects, the *Vg* mRNA level in the dsvg-injected insects decreased by about 90% (Figure 6a). We used *pc3*-specific primers in the qRT-PCR to measure total *pc3* RNA, which contained both *pc3* mRNA and the *pc3* gene, revealing that the *pc3* RNA level decreased by about 79% in the ovariole of the dsvg-injected insect compared with that of the control (Figure 6b). Further, the relative level of *pc3* RNA in other parts of the insect body did not obviously differ among the treatments (Figure 6c), suggesting that the knockdown of *vg* expression by RNA interference specifically affected the accumulation of RSV RNPs in the ovariole of the insect.

**Figure 7:**
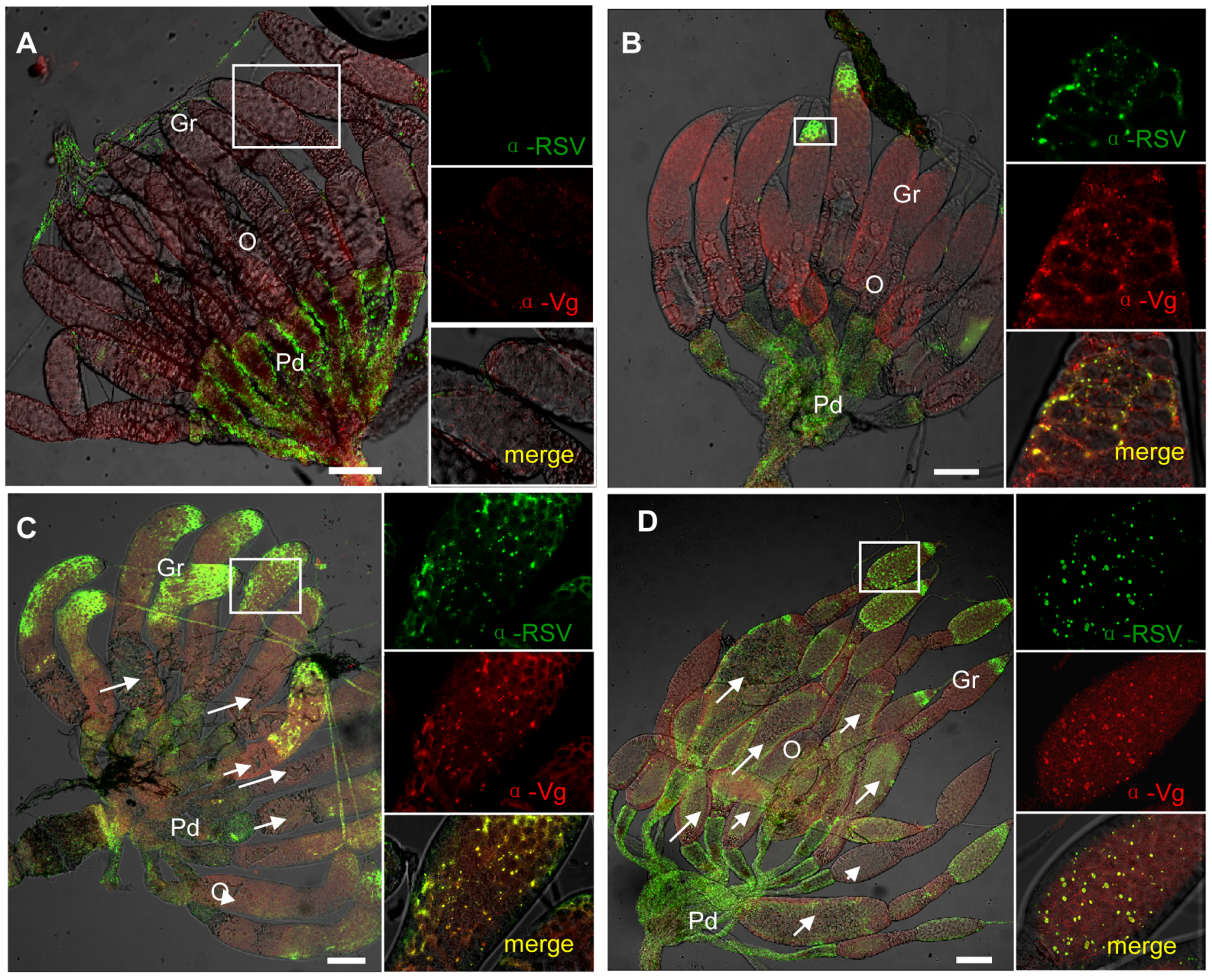
Injection of dsvg or dsgfp resulted in different RSV infection levels in the ovary. Newly emerged adult insects were injected with dsvg or dsgfp, and ovaries were dissected to test RSV or Vg levels 48–c: Ovaries from dsvg-treated insects; d: ovaries from dsgfp-treated insects. a: Type A, ovariole had developed with more than two ooecia, but no Vg accumulated; RSV did not invade the ovariole. b: Type B, Vg had accumulated in the germarium and formed large spots in the tip; RSV began to invade the tip of the germerium of only a few ovarioles. c: Type C, a large amount of Vg had accumulated, forming large spots throughout the germarium, but not in the oocytes at the bottom (white arrows); RSV present in most of the germarium of nearly all ovarioles. d: Type C, Vg had accumulated in the oocyte at the bottom to form yolk globules (white arrows); RSV present throughout the germarium. RSV antibody was conjugated to Alexa Fluor 488. Bar = 100 µm. Gr: germarium, O: oocyte.

To further demonstrate the critical role of Vg in transovarial transmission of RSV, we injected newly emerged adults with dsgfp or dsvg, respectively, and then examined the location of RSV RNPs and Vg in the ovarioles using immunofluoresence. We did not observe differences in the degree of development among the ovarioles dissected from the treated insects, and they had developed more than two ooecia (Figure 7). However, as the expression of Vg was reduced after injected with dsvg, Vg accumulation was also influnced, resulting in no or a small amount of yolk globules present in the oocytes (Figure 7a–c). In most dsgfp treated insects, Vg was highly expressed at 2 days postemergence, the oocytes at the bottom of the egg tube accumulate largely Vg to form yolk globules for their mature (Figure 7d). Besides, because of Vg accumulation, the column of the ooecia was larger, and the ooecia in each ovariole were more distinct than that in the dsvg treated ones. Moreover, the dsvg treated female individuals failed to produce offspring.

The distribution of RSV RNPs in the treated ovarioles could be classified into three types. In the first type (type A), Vg was not transported or just began to be transported into the germarium to form some small spots, while RSV RNPs were only detected in the pedicel and terminal filament but not in the germarium (Figure 7a). In the second type (type B), more Vg accumulated and some formed bigger spots in the anterior part of the germarium, where RSV RNPs existed (Figure 7b). In the third type (type C), a large amount of Vg had accumulated in the germarium, and RSV had invaded most parts of the germarium (Figure 7c, d). Among the insects injected with dsgfp, RSV had generally invaded most part of the ovary and 79% of the ovaries belonged to type C, with only 6% belonged to type A and 15% belonged to type B. In contrast, injection with dsvg obviously affected the entry and subsequent spread of RSV in the insect ovary. As many as 34% and 29% ovaries of insects injected with dsvg belonged to type A and type B respectively, only 37% ovaries belonged to type C (Table 1). All these results indicated that the reduction in Vg expression by RNA interference significantly prevented infection of the insects' ovarioles by RSV.

**Table 1:** Percentage of RSV-infected ovaries with virus in the three location types from insects injected with dsgfp or dsvg dsRNAs.

	Type A^a^		Type B^b^		Type C^c^		Total
dsRNA	*N.*	Percent (%)	*N.*	Percent (%)	*N.*	Percent (%)	*N*.
dsgfp	5	6	13	15	70	79	88
dsvg	30	34	25	29	32	37	87

a : RSV RNPs were only detected in the pedicel and terminal filament but not in the germarium;

b : RSV RNPs were present in the anterior part of germarium;

c : RSV had invaded most parts of the germarium. N: the number of individuals.

## Discussion

Vg, present in the female individuals of most oviparous species and invertebrates, is a member of the large lipid transfer protein superfamily [26], [27]. As the egg yolk precursor protein, Vg is usually synthesized extra-ovarially under hormonal control [28]–[32]. After its synthesis primarily by the fat body of insects, Vg is secreted into the hemolymph, then transported by the circulatory system to the ovary, from where it is absorbed into the growing oocytes via receptor-mediated endocytosis. Once in the growing oocytes, Vg is proteolytically cleaved to generate yolk proteins, lipovitellin and phosvitin, finally serving as nutrients for developing embryos [33]–[36]. Clearly, there is a transovarial transportation system for absorbing nutrients in the oviparous species, including insects.

In this study, we found a high affinity between Vg and RSV pc3, the main component of RSV RNPs (Figure 2). RSV RNPs and Vg might bind together in the hemolymph and then be transported into the ovary of the insect vector via the existing transportation systems for Vg in the female insects. In the immunofluorescence assay, RSV RNPs were only observed in the terminal filaments and pedicels of the ovary before the expression of Vg (Figures 3 and 4). Accompanied by the expression of Vg, RSV RNPs first appeared in the anterior part of germarium at Adult II stage, then spread into most parts of the germarium and the ooecium at Adult III and IV stages (Figure 3). It was obvious that the transovarial transmission of RSV was correlated with the movement of Vg, suggesting that Vg is an important component for RSV to overcome the transovarial transmission barrier.

The development of the insect ovary can be divided into different stages according to ovariole development and morphological characteristics or vitellogenesis, i.e, previtellogenesis, vitellogenesis and postvitellogenesis [37]. Obviously, early development of the ovary is not pertinent to Vg, which is primarily produced and endocytosed into the ovary during vitellogenesis stage [37]. Based on our observations, the RSV RNPs entered the germarium of the insect during an early phase of vitellogenesis (Figure 3a,Adult II).Then it usually invaded all around the germarium, with a few RNPs detected in the oocytes as well (Figure 3a, Adult IV). But the distribution of RSV RNPs in the ovariole of dsvg-injected insects was different. Compared with the control (dsgfp injection), the dsvg injection had obviously affected the accumulation of RSV RNPs in insect ovarioles (Figure 7 and Table 1), although the morphology of the ovarioles was similar, the amount of RSV total *pc3* RNA in the ovary was greatly reduced (Figure 6). These results further indicated that Vg was specifically involved in the invasion of RSV in the insect ovary. As we noted earlier, a large amount of Vg is expressed outside the ovary during vitellogenesis, then enters the ovarioles by endocytosis [38], [39]. RSV RNPs that are bound with Vg via pc3 might be endocytosed in the same way. It is interesting that, even though dsvg injection influenced Vg accumulation and RSV distribution in the ovary, it did not significantly change the development of the ovarioles, which also differentiated several ooecia. Differentiation of the ooecia usually occurs at the previtellogeniesis stage when Vg is not highly expressed. Accumulation of Vg enlarges the column of the ooecia and triggers the maturation of the egg. Thus, lack of Vg may delay the development of the oocytes and can even lead to aplasia [40], [41]; eventually, the dsvg injection caused sterility in the females in our experiment.

Endosymbiotic organisms use different pathways for transovarial transmission. YLSs could pass through the ovariole pedicel and enter the ovary through a deep depression at the posterior pole [13], [42]. Bacteriocytes in insects with telotrophic ovarioles, such as *Nysius ericae*, *Nithecus jacobaeaein*, *Marchalina hellenica*, and *Scaphoideus titanus*, first occur in the germarium and then infect oocytes through the nutritive cord [21], [43], [44]. However, bacteriocytes in *Palaeococcus fuscipennis* move through the intercellular spaces between neighboring follicles, infect the ovary via endocytosis, and then remain on the surface of the oocyte until embryonic development [45], [46]. Cytoplasmic dynein was thought to be an important factor for transovarial transmission of *Wolbachia* in *D. melanogaster* [47]. Obviously, RSV invaded the oocyte neither from the pedicel nor from the filament directly. It is likely that some barriers may exist between the pedicel and the ooecium or between the filament and the germarium, which may prevent the invasion of RSV into the ooecium or germarium (Figures 3a and S3a, c). We first found RSV in the anterior part of the germarium, then in most areas of the germarium and ooecium (Figures 3 and 4). The germarium portion of the ovariole usually contains stem cells, which will further differentiate into oocytes and nurse cells [19], [48]. Some endosymbiotic bacteria, such as *Wolbachia* in *D. melanogaster*, reach oocytes through the stem cells in the germarium [49]. However, for the telotrophic ovarioles of some insects, stem cells will differentiate into both nurse cells on the anterior side of germarium and ooctyes at the base of the germarium [50]–[52]. These oocytes are mainly differentiated from stem cells in the nymph stage, when Vg is not highly expressed. Therefore, despite RSV first invading the germarium at vitellogenesis stage, it does not seem possible that RSV spreads to the oocytes from the stem cells as *Wolbachia* does in *D. melanogaster*. RSV might invade the new nurse cells at the tip of the germarium and then spread throughout the germarium as more nurse cells develop.

Nurse cells of the telotrophic meroistic ovarioles uaually provide nutrients to the oocytes through the nutritive cords [23], [53], [54]. Our study is also the first to find Vg in the nurse cells of telotrophic meroistic ovarioles. After entry into the nurse cells in the germarium, Vg gathered in the yolk globules, which was also seen with electron microscopy (Figure 5a). Immunoelectron microscopy showed that most RSV RNPs accumulated in yolk globules after invading the nurse cells (Figures 5b, c). Then Vg might be further processed into egg yolk at the suspected R/KXXR motif in the vWD domain, the most important site for binding to RSV pc3. Later, RSV might be released from Vg in the nurse cells due to an alteration in the vWD domain, then spread into the oocytes through the nutritive cords (Figure 3c). This scenario could explain why RSV RNPs and Vg did not colocalize together in the ooecium (Figure S3b).

**Figure 8:**
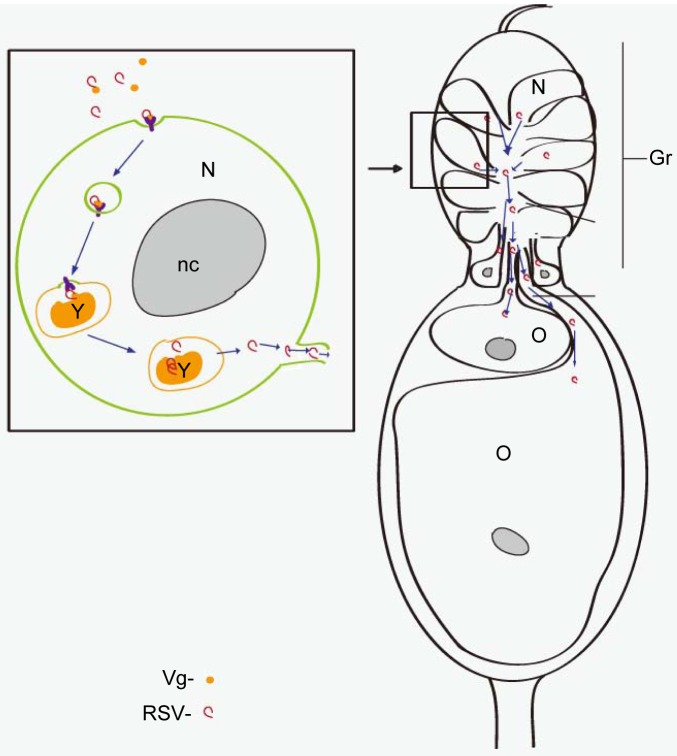
Model of RSV transovarial transmission. RSV binds to Vg outside the ovary and is transported into the germ cells through VgR-mediated endocytosis. RSV first accumulates in yolk granules with Vg and is released into the cytoplasm. RSV finally enters the oocytes through the trophic core, then the nutritive cord. Gr: germarium, NC: nurse cell, Nc: nutritive cord, nc: nucleolus, TC: trophic core, O: oocyte, Y: yolk granule.

Based on all the data obtained, we propose a model for the RSV transovarial transmission in *L. striatellus* (Figure 8). RSV binds to Vg outside the ovariole to form a complex; the complex is then transported into the nurse cells in the germarium via endocytosis. In the nurse cells, Vg is processed into yolk protein to form yolk granules [54]. Most RSV RNPs remain in the yolk granules while some are released into the cytoplasm. Finally, RSV RNPs in the nurse cells spread into the growing oocytes with or without binding with Vg through the nutritive cords. Taken together, RSV can use existing transovarial transport systems in female insects for its own purposes. The model that we propose might also apply to other persistent-propagative animal and plant viruses transmitted by arthropods and perhaps to some bacteriocytes in other insects. Given the implications of the transovarial mechanism for viral evolution and epidemiology, our results urgently call for a broader investigation of this important trait in a wide panel of natural virus–host associations.

## Materials and Methods

### Insect rearing

Individuals of *L. striatellus* used in this experiment were originally captured in Jiangsu Province, China and maintained in the laboratory for nearly 8 years. Both viruliferous and healthy *L. striatellus* were reared separately on rice seedlings grown in glass beakers with soil 1 cm deep. Plants were grown at 25°C with 16 h light/8 h dark in a growth incubator. The insects were transferred to fresh seedlings every 15 days to assure sufficient nutrition. For ensuring that the insects were viruliferous, an individual female insect was fed separately, and the offspring were collected and checked by RT-PCR. The most highly viruliferous colony was kept.

### Yeast two-hybrid assay

Yeast two-hybrid screening was performed using a DUALhunter starter kit (Dualsystems Biotech) according to the manufacturer's protocol. The cDNA library of *L. striatellus* was constructed in prey plasmid pPR3-N using an EasyClone cDNA library construction kit (Dualsystems Biotech), and the bait plasmid was constructed by cloning full-length RSV *pc3* in pDHB1. After library screening, positive clones were selected on SD quadruple-dropout (QDO) medium (SD/-Ade/-His/-Leu/-Trp), and prey plasmids were isolated from these clones for sequencing. To distinguish positive from false-positive interactions and further confirm the interaction of bait and prey proteins, we cotransformed the two plasmids into yeast strain NMY51, and β- galactosidase activity was detected with an HTX Kit (Dualsystems Biotech).

### Chemiluminescent coimmunoprecipitation assay

The chemiluminescent CO-IP assay was carried out according to the manufacturer's instructions (Clontech). Cells of 293FT were cotransformed with pAcGFP1-pc3 and pProLabel-Vg plasmids for 48 h, lysed and incubated with anti-GFP polyclonal antibody (Clontech) for 2 h. Then the lysate was added to protein A/G agarose beads, and the mixture was incubated overnight at 4°C. Beads were collected and washed nine times by centrifugation. Each sample was transferred to a well in a 96-well assay plate. To each well, substrate mix was added, and ProLabel activity was measured at 10-min intervals using a GloMaxTM 96 Microplate Luminometer (Promega).

### Cloning and expression study of *Vg*

Adult insects were ground with a mortar and pestle in liquid nitrogen, and total RNA was isolated following the standard TRIzol reagent (Invitrogen) protocol. The concentration and quality of total RNA were determined using a NanoDrop spectrophotometer (Thermo Scientific), and the cDNA was produced using the Superscript III First Strand Synthesis System (Invitrogen). Based on the *Vg* mRNA sequence published previously [23], *Vg* cDNA was obtained using PCR, 3′ RACE and 5′ RACE (Invitrogen).

To measure Vg expression patterns during the life cycle of *L. striatellus*, we isolated total RNA at different insect life-stages and produced cDNA. Relative *Vg* mRNA levels were measured by real-time PCR with *Vg-* and *ef2*-insect-specific primers following the Light Cycler Taqman Kit (Toyobo) protocol.

### Antibody preparation

RSV RNPs were purified from RSV-infected rice plants and injected into mice to obtain anti-RSV serum. The anti-RSV monoclonal antibody was kindly provided by Xueping Zhou (Zhejiang University). An anti-Vg monoclonal antibody against the Vg peptide CMQQKTKSRSRRS was prepared by Abmart (Shanghai). Vg polypeptides RMQPLNKEEKQNVF, EQKQPNATFKKNIPQR, and RNQQKTKSRSRRS were conjugated to mcKLH and injected into rabbits to make anti-Vg serum.

### GST pull-down assay

RSV *pc3* cDNA fragments were amplified and cloned into PGEX-3X for fusion with glutathione *S*-transferase (GST). The *VitN*, *DUF* or *vWD* and *MBP* cDNA fragments were cloned into pET30a for fusion with his-tag. All recombinant proteins were expressed in *Escherichia coli* strain BL21 and purified. The GST-tag fused protein, RSV pc3-GST, was bound to glutathione-Sepharose beads (GE) for 3 h at 4°C; the mixtures were centrifuged for 5 min at 100×*g* and the supernatants discarded. The His-tag fusion proteins were added to the beads and incubated for 2 h at 4°C. After being centrifuged and washed five times with wash buffer (300 mM NaCl, 10 mM Na_2_HPO_3_, 2.7 mM KCl, and 1.7 M KH_2_PO_4_), the bead-bound proteins were separated by SDS-PAGE gel electrophoresis and detected by western blotting with His-tag antibodies.

### Immunofluorescence microscopy

The anti-RSV monoclonal antibody was labeled with Alexa Fluor 488, and the anti-Vg monoclonal antibody was labeled with Alexa Fluor 594 according to the Alexa Fluor 488 Monoclonal Antibody Labeling Kit and Alexa Fluor 594 Protein Labeling Kit (Invitrogen) instructions, respectively. The ovaries were dissected from *L. striatellus* nymphs and female adults at four stages: 12 h postemergence (Adult I), 24 h postemergence (Adult II), 36 h postemergence (Adult III), and 48 h postemergence (Adult IV) in cold distilled water in a glass plate, and fixed in 4% paraformaldehyde in phosphate-buffered saline (PBS) for 2 h at room temperature. The fixed ovaries were incubated in osmotic buffer (2% triton v/v in PBS) for 4 h at room temperature, and then incubated with labeled anti-RSV and anti-Vg antibody for 2 h at room temperature. The samples were viewed with a laser scanning confocal microscope (Leica TCS SP5) and the images saved by Leica LAS AF Lite.

### Immunoelectron microscopy

Dissected ovaries were fixed for 3 h at 4°C in 4% (v/v) paraformaldehyde and 2.5% (w/v) osmium tetroxide in PBS, and after sequential dehydration in 30%, 50%, 70%, 90%, 95% and 100% alcohol, ovaries were embedded in LR Gold Resin (Fluka Biochemika, Steinheim, Switzerland). Sections of the embedded ovaries were cut at 80 nm, then blocked for 30 min in blocking buffer (goat serum, 1∶100). The blocked sections were incubated at room temperature with the antibodies in order of anti-RSV mouse serum (1∶100) for 2 h, 10-nm gold-conjugated goat-anti-mouse IgG (1∶50, Promega) for 2 h, anti-Vg rabbit serum (1∶100) for 2 h and 15-nm gold-conjugated goat-anti-rabbit IgG (1∶50, Promega) for 2 h with a wash in distilled water after each antibody incubation. They were then stained in 2% neutral uranyl acetate (w/v in distilled water) for 20 min. The sections were viewed with a transmission electron microscope (TEM) at 80 kV accelerating voltage.

### DsRNA injection

Specific *Vg* primers were designed with a 23-nt T7 RNA polymerase promoter DNA fragment on the 5′ end, and the 530-bp *Vg* DNA fragment with the T7 promoter sequence on both sides was amplified by PCR. The PCR products were used to synthesize dsRNA with MEGAscript T7 kit (Ambion). Following the method reported for the brown planthopper [41], we injected fifth instar viruliferous nymphs of *L. striatellus* with 36.8 nl dsvg (2 µg/µl) with dsgfp as the negative control. The newly emerged adults of injected insects were transferred to new culture beakers and allowed to feed on fresh rice seedlings for 48 h, when the ovary should have reached the vitellogenic stage and RSV should be present in large amounts.

### Real-time qRT-PCR

To measure Vg expression levels in *L. striatellus*, we isolated total RNAs from insects at different life-stages and produced cDNAs using the Superscript III First Strand Synthesis System (Invitrogen). Relative *Vg* mRNA levels were measured by real-time PCR with *Vg*-specific primers and *ef2*-insect-specific primers with the Light Cycler Taqman Master kit (Toyobo) according to the manufacturer's instructions. Total RNA was previously isolated from ovaries and the other parts of the insect body that had been injected with dsRNA. After reverse transcription, the RNA level (containing mRNA and genomic RNA) of RSV *pc3*, were measured with specific primers by real-time PCR. The PCR product was detected and analyzed with the Bio-Rad CFX manager.

## Supporting Information

Figure S1 **Confirmed interaction between RSV pc3 and vitellogenin fragment using yeast two-hybrid system and chemiluminescent coimmunoprecipitation.** a: Plasmids were used to co-transform NMY51 yeast cells, which were grown on plates of selective medium SD/-Ade/-His/-Leu/-Trp for 3 days. b: Transformants appear colored in the HTX β-galactosidase assay. 1, Positive transformant of pDHB1-largeT and pDSL-53. 2, Negative transformant of pDHB1-largeT and pPR3N. 3, Transformant of pDHB1-pc3 and pPR3N-Vg. c: AcGFP1-pc3 and Prolabel-vitellogenin were co-transformed and co-expressed in the HEK 293FT cells. The expression of AcGFP1-pc3 was detected with fluorescence microscopy. d: The interaction of the two proteins was examed by ProLabel activity. ProLabel activity of sample (GFP1-pc3 and Label-Vg) and positive (GFP1-53 and Label-T) and negative controls (GFP1-Lam and Label-T) were measured 1 h after addition of substrate.
(PDF)

Figure S2 **Analysis of vitellogenins of** ***Laodelphax striatellu*** **.** Comparison of amino acid sequences of vitellogenin from *L. striatellus* and from *Nilaparvata lugens*.
(PDF)

Figure S3 **Location of RSV in the terminal filament, oocytes and the pedicel.** a: RSV RNPs were present in the pedicel of the ovariole, but not in oocyte, which was linked to the pedicel at the previtellogenesis stage. b: RSV and Vg did not colocalize in the ooecium at the vitellogenesis stage. c: RSV invaded the ovariole from the tip of gemarium. Above the infection site, several layers of cells (denoted by a frame) linked the terminal filament, uninfected by RSV. Anti-RSV and anti-Vg monoclonal antibodies were conjugated to Alexa Fluor 488 and Alexa Fluor 594 separately, bar = 10 µm. TF: terminal filament, Gr: germarium, O: oocyte, Pd: pedicel.
(PDF)
