# Correction: Transovarial Transmission of a Plant Virus Is Mediated by Vitellogenin of Its Insect Vector

**DOI:** 10.1371/journal.ppat.1004141

**Published:** 2014-04-25

**Authors:** The PLOS Pathogens Staff

## Notice of Republication

This article was republished on April 3, 2014, to correct affiliations for all authors and correct legends for Figures 4B and 4C. Please download this article again to view the correct version. The originally published, uncorrected article and the republished, corrected article are provided here for reference.

## Supporting Information

File S1 Originally published, uncorrected article.
(PDF)

File S2 Republished, corrected article.
(PDF)
