# Supplementary figures and images for: Transovarial Transmission of a Plant Virus Is Mediated by Vitellogenin of Its Insect Vector
Source: PLoS Pathog. 2014 Mar 6;10(3):e1003949. doi: 10.1371/journal.ppat.1003949 (PMC3946389; doi:10.1371/journal.ppat.1003949)

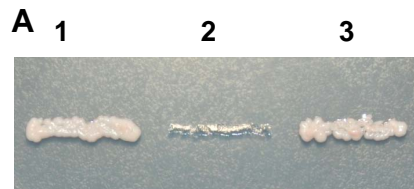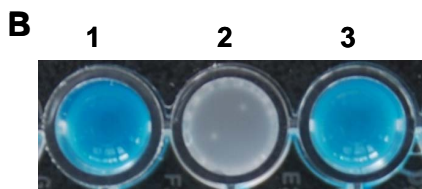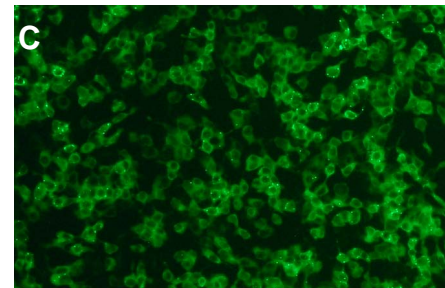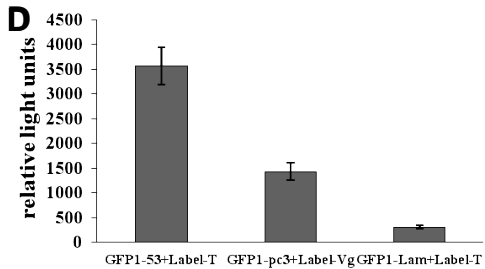

Supplement: Figure S1 — Confirmed interaction between RSV pc3 and vitellogenin fragment using yeast two-hybrid system and chemiluminescent coimmunoprecipitation. a: Plasmids were used to co-transform NMY51 yeast cells, which were grown on plates of selective medium SD/-Ade/-His/-Leu/-Trp for 3 days. b: Transformants appear colored in the HTX β-galactosidase assay. 1, Positive transformant of pDHB1-largeT and pDSL-53. 2, Negative transformant of pDHB1-largeT and pPR3N. 3, Transformant of pDHB1-pc3 and pPR3N-Vg. c: AcGFP1-pc3 and Prolabel-vitellogenin were co-transformed and co-expressed in the HEK 293FT cells. The expression of AcGFP1-pc3 was detected with fluorescence microscopy. d: The interaction of the two proteins was examed by ProLabel activity. ProLabel activity of sample (GFP1-pc3 and Label-Vg) and positive (GFP1-53 and Label-T) and negative controls (GFP1-Lam and Label-T) were measured 1 h after addition of substrate. (PDF) [file ppat.1003949.s001.pdf]

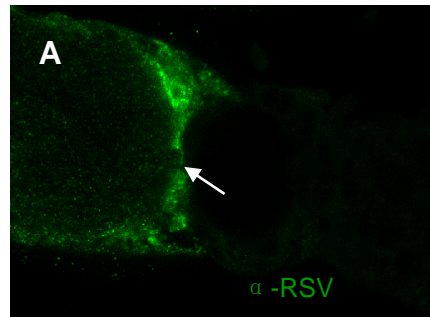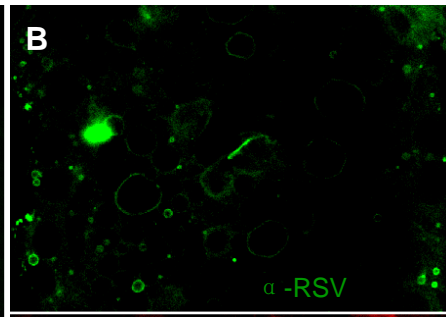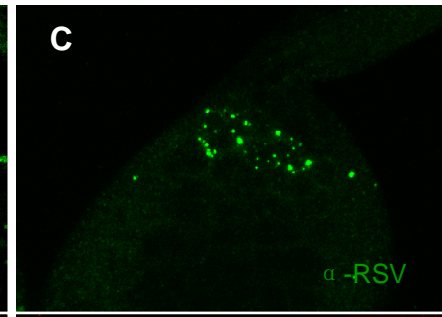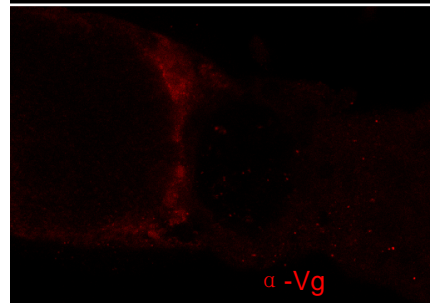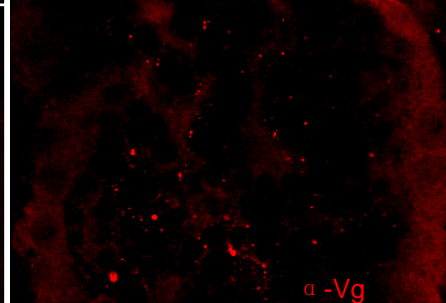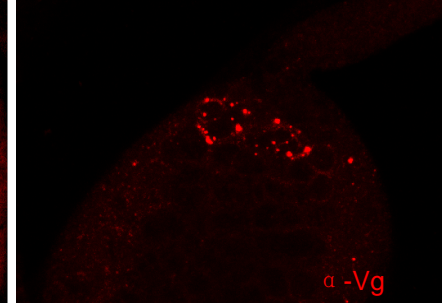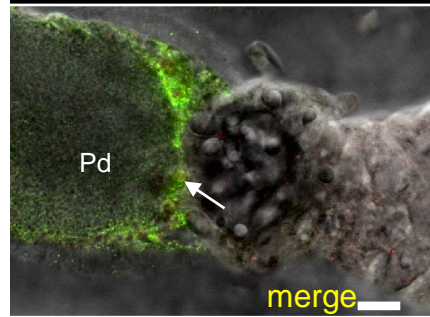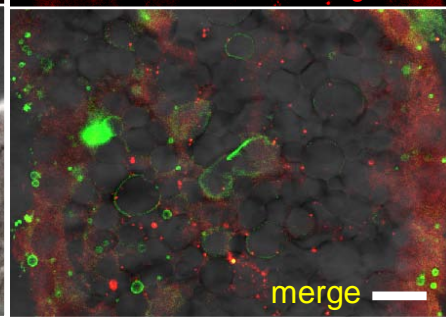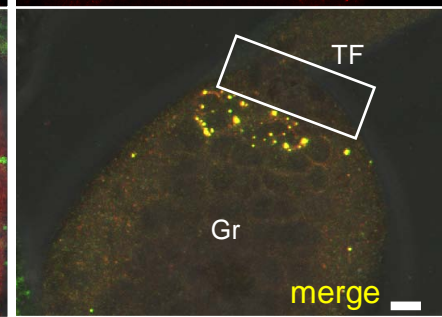

Supplement: Figure S3 — Location of RSV in the terminal filament, oocytes and the pedicel. a: RSV RNPs were present in the pedicel of the ovariole, but not in oocyte, which was linked to the pedicel at the previtellogenesis stage. b: RSV and Vg did not colocalize in the ooecium at the vitellogenesis stage. c: RSV invaded the ovariole from the tip of gemarium. Above the infection site, several layers of cells (denoted by a frame) linked the terminal filament, uninfected by RSV. Anti-RSV and anti-Vg monoclonal antibodies were conjugated to Alexa Fluor 488 and Alexa Fluor 594 separately, bar = 10 µm. TF: terminal filament, Gr: germarium, O: oocyte, Pd: pedicel. (PDF) [file ppat.1003949.s003.pdf]
