# Supplementary material for: Transovarial Transmission of a Plant Virus Is Mediated by Vitellogenin of Its Insect Vector
Source: PLoS Pathog. 2014 Mar 6;10(3):e1003949. doi: 10.1371/journal.ppat.1003949 (PMC3946389; doi:10.1371/journal.ppat.1003949)
Supplement: Figure S2 — Analysis of vitellogenins of Laodelphax striatellu . Comparison of amino acid sequences of vitellogenin from L. striatellus and from Nilaparvata lugens. (PDF) [file ppat.1003949.s002.pdf]

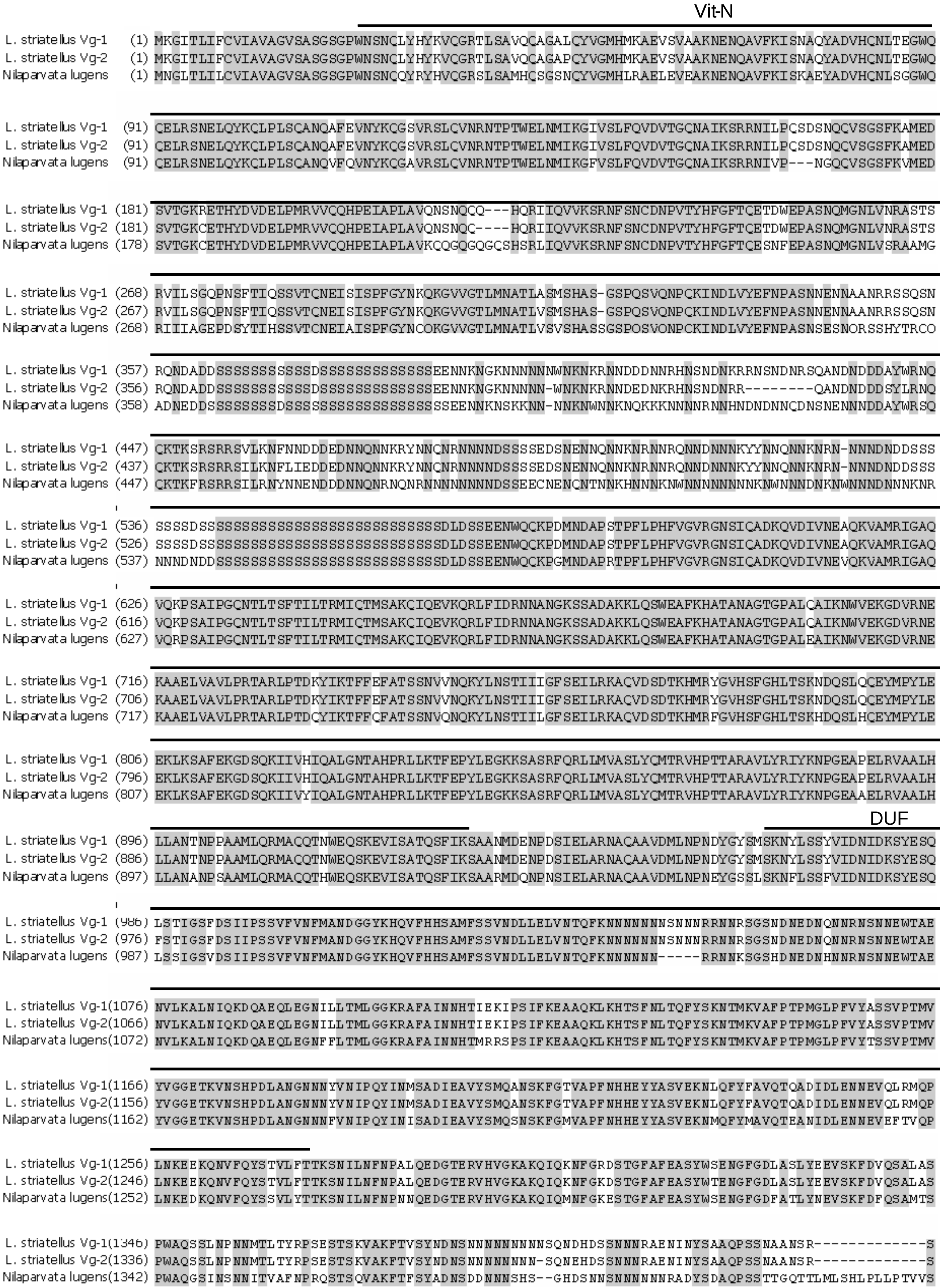
Vit-N
DUF
L. striatellus Vg-1
L. striatellus Vg-2
Nilaparvata lugens

```
L. striatellus Vg-1(1423) RQNEFLHKVASGISGADAMVVDVSAEFQGSHGQSSAQYVATLAMANSDASPNARMLFFASMDPANSGSKAQVCAAAASHFPNVPLMNFND
L. striatellus Vg-2(1412) RQNEFLHKVASGISGADAMVVDVSAEFQGSHGQSSAQYVATLAMANSDASPNARMLFFASMDPANSGSKAQVCAAAASHFPNVPLMNFND
Nilaparvata lugens(1430)  RQNEFLRKAAAGISGADAMVVDVSARFQDSHGQSNAQYVATVAMANSDASPNARMLFFASMNPANSDSKAQVCAAAASNFPNVPLMNFHD

L. striatellus Vg-1(1513) ALKANPDSHITADIAFGEKCNAGGHIRADAKMSQTQEFQDFAKNRPMAKKCFQLMQQGQALEYACQNATKVANMLNNYEVSLKYDRVPNA
L. striatellus Vg-2(1502) ALKANPDSHITADIAFGEKCNAGGHIRADAKMSQTQEFQDFAKNRPMAKKCFQLMQQGQALEYACQNATKVANMLNNYEVSLKYDRVPNA
Nilaparvata lugens(1520)  ALKANPTSRISADIAFGAQCNAGGHIHADAKLSQTQEFQEYAKSRPMAKKCFQLMEKGQALEYACQNATKVANMLNNYEVSVKYDRVSSV

L. striatellus Vg-1(1603) LKNATYNIYSALAQVAFPYLSENMFSQHSNPAGKIDLNARFNYMLRYFNASINSPFFTANFKNVEVNPAVRPLVIFHPSLNSLELMSYNE
L. striatellus Vg-2(1592) LKNATYNIYSALAQVAFPYLSENMFSQHSNPAGKIDLNARFNYMLRYFNASINSPFFTANFKNVEVNPAVRPLVIFHPSLNSLELMSYNE
Nilaparvata lugens(1610)  FKNVTYSIYSALAQAAYPYHNENMFSQNSNPSGKIDLNARFNYMLRYFNASINTPFFSANVKNVEVHHALRPLVIFHPSLNSLEHLSSCP

                                                                                        vWD
L. striatellus Vg-1(1693) NYDYPTCS---VSKNSISTFDNKTYSADLEGWHVMFASTPKNYNDNSGRYSASNSQSNSFYKYKKVAILAKNSGSQRKAVKMLLGDNVID
L. striatellus Vg-2(1682) NYDYPTCS---VSKNSISTFDNKTYSADLEGWHVMFASTPKNYNDNSGRYSASNSQSNSFYKYKKVAILAKNSGSQRKAVKMLLGDNVID
Nilaparvata lugens(1700)  TMRTTITQHVLSARTPSAHSTTRPTHADLEGWHVMFASTPKNFNDNSGRYSASNSQSNSFYKYKKVVVLAKNAGSQRKAVKMLLGENVID

L. striatellus Vg-1(1780) ITPSGSESNNNSPNANVQVNGNKMHIANNRLASFEDFDGETLVEISVNDNGEVQVQSPSHGIAVNHDGANFMIDADSYYRGEVRGLCGTY
L. striatellus Vg-2(1769) ITPSGSESNNNSPNANVQVNGNKMHIANNRLASFEDFDGETLVEISVNDNGEVQVQSPSHGIAVNHDGANFMIDADSYYRGEVRGLCGTY
Nilaparvata lugens(1790)  INPSGSESSDNSPNANVQVNGNKVQIANNRMASFDDFDGETLVEISVTDNGEVQVQSSSHGIAVYHDGANFIIDADSYHRGEVRGLCGTY

L. striatellus Vg-1(1870) SGDKYTDFTTPK-KCVLREAKLFAATYALPGSSNSNVEQLKRQADQVSCFKRHEILADVITSNDYDRSSSSSSNQRNNKNNNNNNNRYNK
L. striatellus Vg-2(1859) SGDKYTDFTTPK-KCILREAKLFAATYALPGSSNSNVEQLKRQADQVSCFKRHEILADVITSNDYDRSSSSSSNQRNNKNNNNNN-RYNK
Nilaparvata lugens(1880)  SGDKYSPLHPEKNKCIMREAIPFAATYALPGSSNSNVEQLKRQADQMTCFRRRHIFANVITSNDYDRSSSSSSSNRNNNRNNNKN-----

L. striatellus Vg-1(1959) NSSMKYQTSDSSSSTDLIQDIKNNGDHVCFSIRPIPKCQHGSSPAGSSEKEVQYLCISQGKNADYWAEQIRSGRYVNLEQKQPNATFKKN
L. striatellus Vg-2(1947) NSSMKYQTSDSSSSTDLVQDIKNNGDHVCFSIRPIPKCQHGSSPAGSSEKEVQYLCISQGKNADYWAEQIRSGRYVNLEQKQPNATFKKN
Nilaparvata lugens(1965)  NRSNNNSSERLANPTKLIQDVKNNGDQVCISIRPVPKCQKGFSPAGSSEKEVDYVCMSHGKNAQFWINQIFQGGYVLLEQKQHNATFMKN

L. striatellus Vg-1(2049) IPQRCVRDN
L. striatellus Vg-2(2037) IPQRCVRDN
Nilaparvata lugens(2055)  IPQRCVRDN
```
